# Risk Factors and Prediction of Acute Kidney Injury in Hospitalized Urology Patients: A Retrospective Cohort Study

**DOI:** 10.3390/jcm15093495

**Published:** 2026-05-02

**Authors:** Nomy Levin Iaina, Hesham Elshami, Murad Asali

**Affiliations:** 1 Department of Nephrology and Hypertension, Barzilai University Medical Center, Ashkelon 7830604, Israel; 2 School of Medicine, Faculty of Health Sciences, Ben Gurion University of the Negev, Be’er Sheva 8410501, Israel; 3 Department of Anesthesiology, Barzilai University Medical Center, Ashkelon 7830604, Israel; 4 Department of Urology, Barzilai University Medical Center, Be’er Sheva 8430905, Israel; murada@bmc.gov.il

**Keywords:** acute kidney injury, urology ward, hospitalized urology patients, monitored inpatient cohort, risk stratification

## Abstract

**Background/Objectives:** Acute kidney injury (AKI) is a clinically important complication among hospitalized urology patients. However, data from general urology inpatient populations remain limited. We aimed to assess AKI frequency in a monitored urology inpatient cohort, identify associated predictors, and develop an exploratory admission-based risk stratification model. **Methods**: We conducted a retrospective observational cohort study of adults admitted to a tertiary urology ward between June 2023 and May 2024 who had at least two serum creatinine measurements during hospitalization. AKI was defined according to Kidney Disease: Improving Global Outcomes (KDIGO) serum creatinine criteria. Demographic, clinical, laboratory, and procedural data were analyzed. Multivariable logistic regression identified factors associated with AKI and was used to construct a reduced exploratory admission-based risk model. **Results**: Among 196 monitored patients, 67 (34.2%) developed AKI during hospitalization, and 82.1% had KDIGO Stage 1 AKI. Higher admission serum creatinine, hypertension, nephrolithiasis, and ureteral interventions were independently associated with AKI. AKI was also associated with longer hospitalization (6.4 ± 4.2 vs. 5.1 ± 3.2 days, *p* = 0.044). The reduced exploratory model identified low-, intermediate-, and high-risk groups with progressively increasing AKI incidence (7.7%, 32.3%, and 76%, respectively; AUC = 0.76). **Conclusions**: In this monitored cohort, AKI was frequent and associated with admission characteristics and prolonged hospitalization. These findings support targeted renal monitoring in higher-risk patients. The admission-based risk model is exploratory and requires validation in prospective multicenter cohorts before clinical implementation.

## 1. Introduction

Acute kidney injury (AKI) represents a major global public health challenge and is associated with substantial morbidity, mortality, and healthcare utilization [1]. It affects approximately 5% of hospitalized patients and more than 25% of critically ill individuals and is increasingly recognized as an important contributor to long-term kidney dysfunction and adverse cardiovascular outcomes [1,2]. Even transient episodes of AKI have been linked to increased short- and long-term mortality [2,3]. Despite advances in clinical care, the incidence of AKI among hospitalized patients continues to pose a major clinical burden, driven by aging populations, increasing comorbidity burden, and expanding use of invasive procedures and nephrotoxic therapies [1].

The pathophysiology of AKI involves complex interactions among hemodynamic instability, ischemic injury, inflammatory activation, endothelial dysfunction, and tubular cell damage [1]. Established risk factors include advanced age, pre-existing chronic kidney disease (CKD), hypertension, diabetes mellitus, cardiovascular disease, hypovolemia, sepsis, and exposure to nephrotoxic agents [1,2]. The Kidney Disease: Improving Global Outcomes (KDIGO) guidelines emphasize the importance of early recognition based on dynamic changes in serum creatinine and urine output and highlight the prognostic significance of even mild renal impairment [4].

In urology wards, AKI frequently arises from distinct and potentially reversible mechanisms, including urinary tract obstruction, peri-procedural ischemia, contrast exposure, postoperative complications, and infectious processes. Surgical and endoscopic interventions such as ureteroscopy, nephrectomy, and urinary diversion have been associated with increased AKI risk, particularly among patients with limited renal reserve [5,6,7]. Previous studies in urologic and major non-cardiac surgical populations have reported AKI incidence rates ranging from 9% to 15%, with baseline renal dysfunction and procedural complexity emerging as key predictors [7,8,9,10]. However, available data have focused predominantly on selected procedural or postoperative populations, and data on AKI in general urology inpatient populations, including both surgical and non-surgical admissions, remain limited.

The primary objective of this study was to determine the incidence and clinical predictors of AKI among hospitalized urology patients in a tertiary care center. The secondary objectives were to evaluate the impact of AKI on short-term clinical outcomes and to develop an exploratory admission-based risk stratification model for early identification of high-risk patients.

## 2. Materials and Methods

### 2.1. Study Design and Setting

This retrospective observational cohort study was conducted in the Department of Urology at Barzilai University Medical Center, a tertiary referral hospital in Ashkelon, Israel. The study period extended from 1 June 2023 to 31 May 2024. Reporting of the study was revised in accordance with the STROBE recommendations for observational studies [11], and a completed STROBE checklist is provided as Supplementary File S1.

### 2.2. Study Population and Recruitment

All consecutive adult patients aged 18 years or older who were admitted to the urology ward during the study period were screened for eligibility using the hospital electronic medical record (EMR) and laboratory database.

### 2.3. Eligibility Criteria

Patients were included if they met all of the following criteria: hospitalization in the urology ward during the study period, age of hospitalization of ≥ 18 years, length of stay of at least 72 h, and availability of at least two serum creatinine measurements obtained at least 24 h apart during hospitalization. Patients were excluded if they had a hospital stay shorter than 72 h, fewer than two creatinine measurements, chronic dialysis treatment, or prior kidney transplantation. The requirement for serial creatinine measurements was intended to enable assessment of dynamic kidney function changes according to creatinine-based AKI criteria. A minimum hospital stay of 72 h was required to allow sufficient time for repeated biochemical assessment and ascertainment of AKI during hospitalization.

Because serum creatinine monitoring during hospitalization was clinician-driven rather than protocolized, the final analytic cohort represents a monitored subgroup of urology inpatients rather than the entire population of ward admissions. Accordingly, incidence estimates reported in this study apply to this monitored analytic cohort and should not be interpreted as population-level estimates for all urology admissions.

### 2.4. Data Collection

Demographic, clinical, laboratory, and procedural data were extracted retrospectively from the EMR. Variables collected included age, sex, baseline comorbidities, admission diagnosis, vital signs at admission, procedural and surgical interventions during hospitalization, serum creatinine measurements, length of stay, discharge destination, and in-hospital outcomes. Only variables that were available in a structured and sufficiently reliable form in the medical record were included in the primary analysis. AKI etiology was not formally adjudicated. Data on nephrotoxic medication exposure, contrast administration, fluid balance, and infection severity were not collected in the retrospective dataset and therefore were not included in the present analysis.

### 2.5. Definition of Acute Kidney Injury

AKI was defined according to the Kidney Disease: Improving Global Outcomes (KDIGO) serum creatinine criteria [4]. Urine output data were not consistently available and were therefore not incorporated into the AKI definition. Baseline creatinine was operationally defined as the admission serum creatinine value because reliable pre-admission outpatient creatinine measurements were not consistently available.

AKI was diagnosed when either of the following occurred during hospitalization: (1) an increase in serum creatinine of at least 0.3 mg/dL within 48 h, or (2) an increase in serum creatinine to at least 1.5 times the baseline value within 7 days. Because admission creatinine may already have been elevated in some patients, particularly in obstructive or CKD-related presentations, this approach may have led to misclassification in patients presenting with community-acquired AKI or AKI superimposed on CKD. Accordingly, AKI classification was based on dynamic in-hospital creatinine changes and should be interpreted in that context.

### 2.6. Outcomes

The primary outcome was the occurrence of AKI during hospitalization. Secondary outcomes were length of hospital stay, discharge destination, and need for KRT.

### 2.7. Statistical Analysis

Continuous variables were presented as mean ± standard deviations (SDs) or median and interquartile range, as appropriate. Between-group comparisons were performed using Student’s *t*-test or the Mann–Whitney U test for continuous variables and the chi-square test or Fisher’s exact test for categorical variables. Multivariable logistic regression was used to identify factors independently associated with AKI. Candidate variables for multivariable modeling were selected from the available dataset based on clinical relevance and univariable associations. Odds ratios (ORs) with 95% confidence intervals were reported. Reference categories for categorical variables were predefined. Model discrimination was assessed by the area under the receiver operating characteristic curve, and calibration was assessed using the Hosmer–Lemeshow goodness-of-fit test.

Because length of stay (LOS) was right-skewed, supplementary and sensitivity analyses were performed. A severity-oriented analysis compared patients with Stage 1 AKI, patients with more severe AKI, and patients without AKI. Additional sensitivity analyses were undertaken to examine whether the association between AKI and LOS was influenced by baseline renal dysfunction, including a restricted analysis limited to patients with normal admission creatinine (≤1.2 mg/dL).

Based on the fitted model, predicted probabilities were calculated for each patient and used to assign patients to pre-specified low-risk, intermediate-risk, and high-risk categories (<20%, 20–50%, and >50%, respectively). For the reduced exploratory model, variables were intentionally limited to a small number of immediately available admission measures, to preserve parsimony, bedside applicability, and to reduce the risk of overfitting in this single-cohort dataset. These thresholds were selected a priori for clinical interpretability and were not optimized according to model performance. Because the model was derived and evaluated within the same cohort, and no internal resampling or external validation was performed, the prediction model should be regarded as exploratory and hypothesis-generating rather than ready for clinical implementation. All analyses were performed using Python 3.11, and a two-sided *p* value < 0.05 was considered statistically significant.

### 2.8. Ethical Approval

The study was conducted in accordance with the Declaration of Helsinki and was approved by the Institutional Review Board of Barzilai University Medical Center (approval number 0057-24-BRZ, 18 July 2024). The requirement for informed consent was waived because of the retrospective design and use of routinely collected data, in accordance with local regulations.

### 2.9. Artificial Intelligence Assistance

Portions of the manuscript were edited for language clarity and grammatical accuracy using ChatGPT (version 5.2, OpenAI). The tool was used exclusively for linguistic refinement. All scientific content, data interpretation, and conclusions are the sole responsibility of the authors.

## 3. Results

### 3.1. Study Population

**Figure 1:**
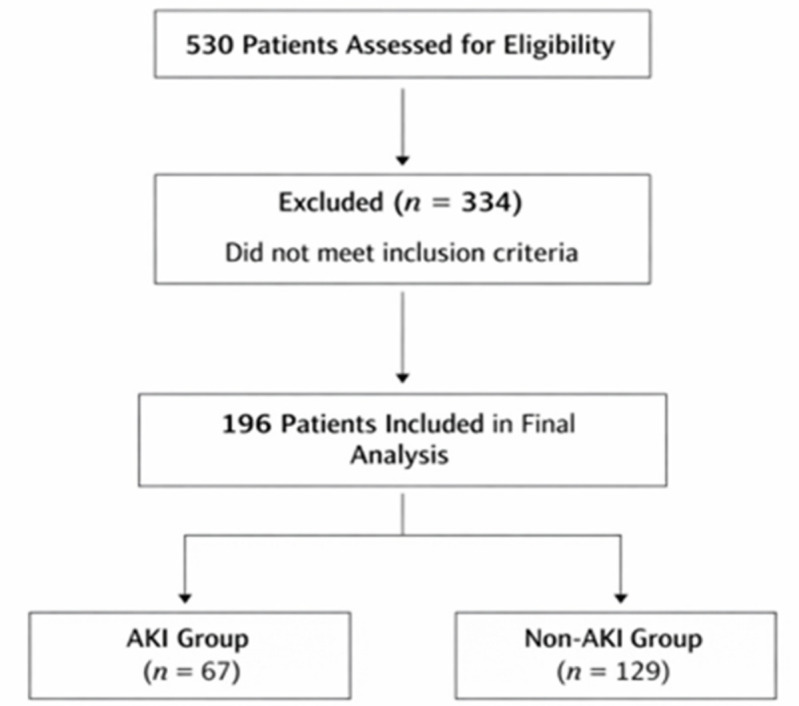
Study Flow Diagram. Flow chart showing patient screening, exclusion based on predefined eligibility criteria, and final study cohort.

During the study period, 530 adult patients were admitted to the urology ward. Of these, 196 patients met the inclusion criteria and had at least two serum creatinine measurements during hospitalization and were included in the final analysis. The screening and selection process is summarized in Figure 1. Serum creatinine monitoring during hospitalization was clinician-driven and preferentially performed in patients perceived to be at increased clinical risk, resulting in non-uniform surveillance across the study population.

All incidence estimates reported in this study therefore apply to this monitored subgroup rather than to the entire urology inpatient population. Baseline data for non-included admissions were not available in a sufficiently structured form to permit a formal comparison between included and non-included patients; therefore, the extent of surveillance-related selection bias could not be directly quantified. Among the included patients, 67 (34.2%) developed AKI during hospitalization.

### 3.2. Baseline Characteristics

Baseline demographic and clinical characteristics of patients with and without AKI are presented in Table 1. Patients who developed AKI were older on average than those without AKI; however, this difference did not reach statistical significance. AKI occurred significantly more frequently in male patients and in those with a history of hypertension. Diabetes mellitus was more frequent in the AKI group, although the difference did not reach statistical significance. No statistically significant differences were observed between groups with regard to dyslipidemia, congestive heart failure, or malignancy.

### 3.3. Causes of Admission and In-Hospital Procedures

**Figure 2:**
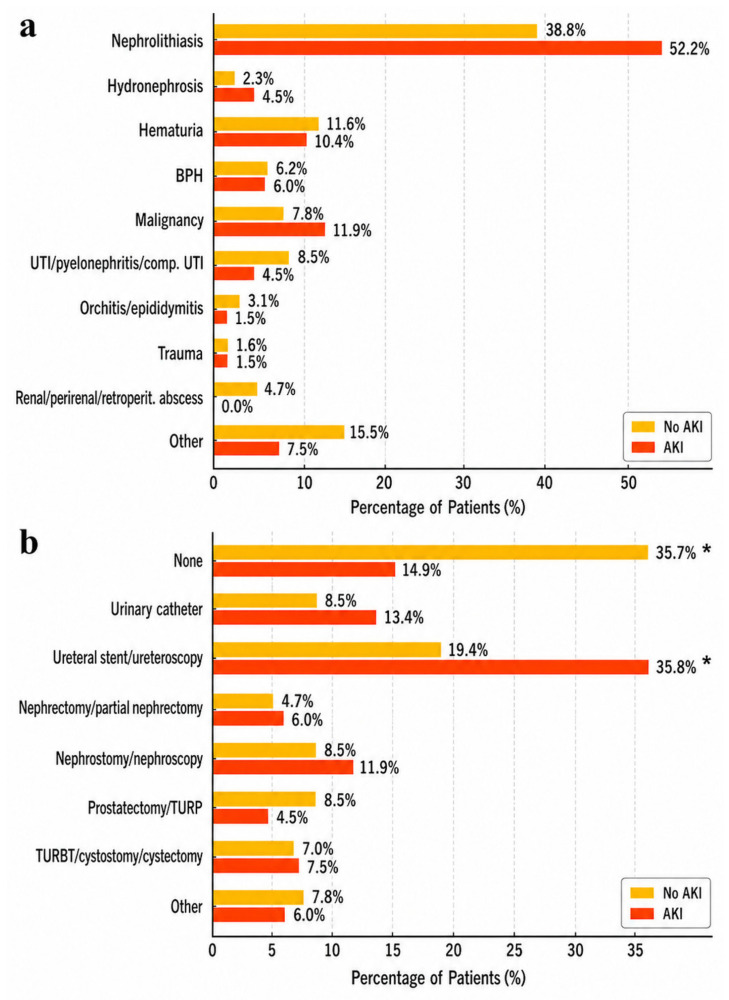
Admission diagnoses, procedures and surgeries performed during hospitalization in patients with and without AKI. (**a**) Primary admission diagnoses among patients with and without AKI. Although kidney/urinary tract stones and malignancies were more common in patients with AKI, the differences were not statistically significant. (**b**) Procedures and surgeries performed during hospitalization in patients with and without AKI. Ureteral stent placement and ureteroscopy were significantly more common in patients who developed AKI, whereas non-interventional management was more frequent among those without AKI. AKI = acute kidney injury; * *p*-value = 0.045 for X^2^ comparison between AKI and non-AKI groups. A *p*-value < 0.05 was considered statistically significant.

Reasons for hospitalization and in-hospital procedures are summarized in Figure 2. Nephrolithiasis and malignancy were more frequent causes of admission among patients who developed AKI, although these differences were not statistically significant. Overall, the distribution of admission diagnoses did not differ significantly between patients with and without AKI (*p* = 0.33). In contrast, significant differences were observed in the types of procedures performed during hospitalization (*p* = 0.045). Ureteral stent placement and ureteroscopy were more common among patients who developed AKI, whereas the absence of any interventional procedure was more frequent among patients without AKI.

### 3.4. Serum Creatinine Trends and AKI Severity

**Figure 3:**
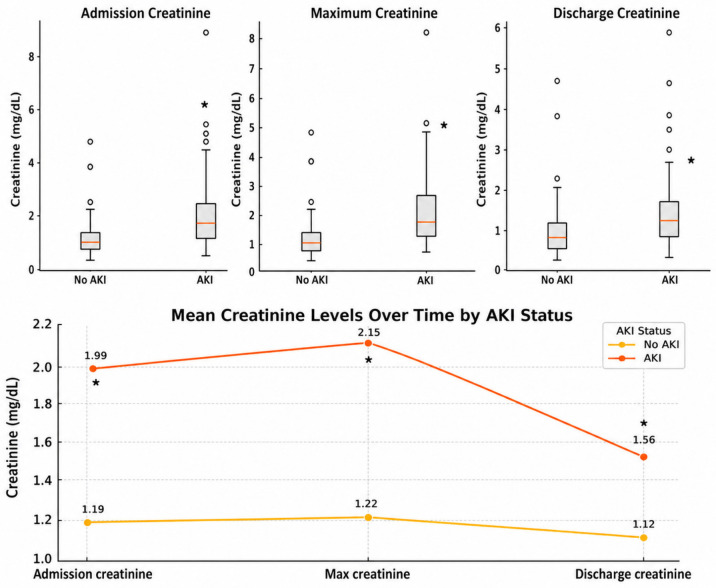
Serum creatinine levels during hospitalization in patients with and without AKI. Patients who developed AKI had significantly higher creatinine levels at all time points. Although kidney function improved by discharge, creatinine remained elevated compared to patients without AKI. * p-values < 0.05 for all comparisons between AKI and non-AKI groups.

Patients who developed AKI had significantly higher serum creatinine levels at admission, higher creatinine peak values during hospitalization, and higher discharge creatinine levels compared with patients without AKI (Figure 3). Although kidney function improved by discharge in most AKI patients, creatinine levels remained significantly higher than in patients without AKI. Male patients had higher creatinine levels than female patients in both AKI and non-AKI groups. In both genders, creatinine levels typically peaked during hospitalization and declined before discharge (Figure 3).

Based on KDIGO criteria, most AKI cases were classified as Stage 1. Specifically, 55 patients (82.1%) had Stage 1 AKI, 5 patients (7.5%) had Stage 2 AKI, and 7 patients (10.4%) had Stage 3 AKI. Of those patients with Stage 1 AKI, 48 patients (88%) met the absolute creatinine change criterion of ≥0.3 mg/dL relative to admission creatinine, whereas 7 patients (12%) met the relative criterion of ≥1.5 and <2 times of baseline.

**Figure 4:**
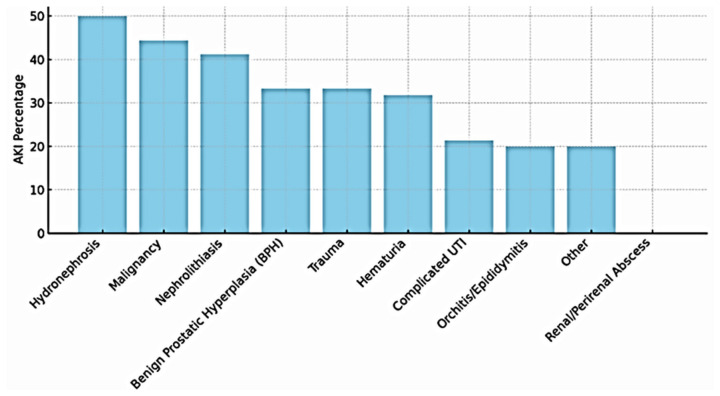
Incidence of AKI by primary reason for hospitalization. The highest AKI rates were observed in patients admitted with hydronephrosis, malignancy, or urinary tract stones, while the lowest rates were seen in those with urinary or testicular infections and abscesses. Most AKI cases were clinically attributed to postrenal mechanisms and were acknowledged by the treating urology team. AKI = acute kidney injury.

The highest AKI incidence was observed among patients with hydronephrosis (50%), malignancy (44.4%), and nephrolithiasis (41.2%), whereas the lowest incidence was observed among patients admitted for infectious conditions (Figure 4). Most AKI cases were clinically attributed to post-renal mechanisms and were clinically recognized and managed by the treating teams.

### 3.5. Clinical Outcomes

Patients who developed AKI had a significantly longer length of hospital stay (LOS) than patients without AKI. Mean LOS was 6.4 ± 4.2 days in the AKI group versus 5.1 ± 3.2 days in the non-AKI group (*p* = 0.044). Median LOS was 5 days (IQR 3–8) in the AKI group and 4 days (IQR 2–6) in the non-AKI group. This difference remained statistically significant on non-parametric testing (Mann–Whitney U test, *p* = 0.047). Because LOS distribution was right-skewed, we also performed sensitivity analyses. We identified one extreme LOS value in the non-AKI group (49 days). After exclusion of this outlier, the between-group difference remained statistically significant (*p* = 0.042).

We also performed a supplementary severity-oriented analysis within the AKI cohort. Patients in the Stage 1 AKI subgroup had a median LOS of 5 days (IQR 3–8), whereas the remaining patients with more severe AKI had a median LOS of 10 days (IQR 6–17). The latter group had significantly longer LOS both compared with the Stage 1 AKI subgroup (*p* = 0.033) and compared with patients without AKI (*p* = 0.011). There was also a significant difference between the Stage 1 AKI subgroup and the non-AKI group (*p* = 0.041).

We performed additional sensitivity analyses to further assess whether the association between AKI and prolonged hospitalization was influenced by baseline renal dysfunction. In a restricted analysis limited to patients with normal admission creatinine (≤1.2 mg/dL), AKI remained associated with longer hospitalization (median 6 [IQR 4–8] vs. 4 [IQR 3–6] in AKI vs. non-AKI patients, respectively; *p* = 0.015). In a multivariable LOS model adjusted for admission creatinine, AKI was associated with an approximately 1.17-day longer hospitalization (*p* = 0.053). In a sensitivity analysis excluding the extreme LOS outlier in the non-AKI group, the adjusted association was significant, with an estimated 1.23-day longer hospitalization (*p* = 0.036).

No significant differences were observed in discharge destination between groups, with more than 95% of patients in both groups discharged home. No patients required KRT, and no in-hospital deaths were recorded.

### 3.6. Risk Prediction Model

Multivariable logistic regression analysis was performed to identify factors independently associated with AKI. The results of the full multivariable model are presented in Table 2. In the full multivariable model, higher admission serum creatinine, ureteral stent placement or ureteroscopy, hypertension, and nephrolithiasis were independently associated with higher odds of AKI during hospitalization. Ureteral stent placement and ureteroscopy were analyzed as a combined procedural category for pragmatic modeling purposes; this grouping was intended to represent endourologic/ureteral intervention rather than to imply identical mechanisms of kidney injury. Admission serum creatinine was the strongest predictor; each 1 mg/dL increase was associated with an approximately threefold increase in the odds of AKI. Male sex and age were included in the multivariable model but were not independently associated with AKI.

The reduced exploratory model was intentionally limited to a small number of immediately available admission variables to preserve parsimony and bedside applicability and to reduce the risk of overfitting in this single-cohort dataset.

To support early clinical risk assessment, we developed a reduced exploratory admission-based risk model using admission serum creatinine and history of hypertension. These variables were selected because they were available at the time of admission and provided a simple clinically applicable framework for early risk stratification. The corresponding logistic regression formula was as follows (where Cr = admission creatinine and HTN = history of hypertension [1 = yes, 0 = no]):

logit = −2.546393 + 1.006557 × Cr + 0.774915 × HTN

Predicted probabilities were calculated for each patient, and patients were categorized into prespecified low-risk (<20%), intermediate-risk (20–50%), and high-risk (>50%) groups.

We then assessed the observed distribution of AKI events across the risk categories generated by the reduced admission-based model. Fifty-five patients (28.1%) were classified as low risk, 104 (53.1%) as intermediate risk, and 37 (18.9%) as high risk for AKI development. The distribution of observed AKI events differed significantly across these categories (χ^2^ = 27.49, *p* < 0.0001), with AKI incidence increasing from 7.7% in the low-risk group to 32.3% in the intermediate-risk group and 76.0% in the high-risk group. The reduced model demonstrated reasonable discrimination (AUC = 0.76), with an overall accuracy of 74.5%.

## 4. Discussion

### 4.1. Principal Findings

In this retrospective cohort study of hospitalized urology patients, we observed a high incidence of AKI, affecting more than one-third of patients within the monitored subgroup who underwent serial creatinine assessment during hospitalization. This estimate should not be interpreted as reflecting the incidence of AKI across all urology admissions, as surveillance was non-uniform and preferentially performed in patients perceived to be at higher clinical risk.

Most AKI events were classified as KDIGO Stage 1, while moderate and severe cases were less frequent. A more granular descriptive analysis of patients with mild creatinine-based AKI patterns showed that most such cases in our cohort were identified based on an absolute creatinine change of at least 0.3 mg/dL, whereas a smaller proportion met the relative threshold of 1.5 times baseline. These findings suggest that the predominance of Stage 1 AKI in our cohort was largely driven by modest but clinically meaningful creatinine changes.

AKI development was independently associated with elevated admission serum creatinine, hypertension, nephrolithiasis, and ureteral interventions. In addition, AKI was associated with prolonged hospitalization, despite the absence of dialysis requirement or in-hospital mortality. These findings highlight that AKI is a frequent and clinically relevant complication in urology wards, including among patients without critical illness. Importantly, supplementary analyses showed that even Stage 1 AKI was associated with longer LOS compared with patients without AKI, supporting the clinical relevance of mild kidney injury.

AKI is increasingly recognized as a heterogeneous and multifactorial syndrome rather than a single uniform entity. In most clinical settings, management remains centered on early recognition, treatment of the underlying cause, prevention of further injury, and supportive care, rather than on a specific disease-modifying therapy [1]. In this context, our findings should be interpreted primarily as supporting improved risk awareness and targeted monitoring in hospitalized urology patients, rather than as establishing a validated interventional pathway.

### 4.2. Comparison with Previous Studies

Direct comparison of the AKI incidence observed in our cohort with previously published studies should be made with caution. Much of the existing literature in urology has focused on selected postoperative or procedure-specific populations, including nephrectomy cohorts, radical cystectomy cohorts, and more recently prospective endourologic cohorts undergoing retrograde intrarenal surgery [5,6,7,8,9,12].

By contrast, the present study evaluated a monitored general urology inpatient cohort that included both surgical and non-surgical admissions. This distinction is important, as AKI risk in general urology inpatients may reflect a broader and more heterogeneous combination of mechanisms, including urinary obstruction, malignancy, baseline renal vulnerability, infection-related factors, and procedural exposures. Our study therefore extends the current literature by addressing AKI in a broader real-world urology ward population rather than in a single procedural subgroup.

Consistent with this difference in case-mix, the AKI incidence observed in our monitored cohort was higher than rates reported in several procedure-specific studies, which have generally described AKI in more selected perioperative or endourologic settings, ranging from 9% to 15% [5,9,12]. For example, in a recent prospective multicenter study of patients undergoing retrograde intrarenal surgery, Candela et al. reported an AKI incidence of 6.2%, with all events classified as KDIGO Stage 1, highlighting the comparatively selected and procedure-specific nature of that cohort [7].

This distinction is also clinically important because AKI in general hospitalized patients often reflects multiple overlapping mechanisms and varying trajectories of injury and recovery. Contemporary reviews emphasize that, despite major advances in AKI pathophysiology, bedside management in most settings still depends largely on timely recognition, careful clinical monitoring, and supportive prevention strategies [1]. Our findings therefore align more closely with this broader clinical framework than with a procedure-specific complication model alone.

The higher incidence in the present study may reflect broader inclusion of non-surgical admissions, systematic creatinine-based surveillance, and application of KDIGO criteria, which enable detection of milder forms of AKI. Similarly, large postoperative cohorts and consensus reports have demonstrated AKI rates below 15% following major abdominal and urologic procedures, particularly when systematic perioperative monitoring and supportive care are implemented [6,8,9].

Consistent with prior literature, baseline renal dysfunction emerged as the strongest predictor of AKI [1]. Elevated admission creatinine reflects reduced renal reserve and increased vulnerability to acute insults [1,3]. Hypertension was also associated with increased AKI risk, in line with previous evidence linking chronic vascular injury to impaired renal adaptive capacity [2,3]. Moreover, systematic review and consensus statements have similarly identified hypertension and comorbidity burden as consistent risk factors for post-operative AKI [1,7], further supporting our observation. However, these associations have rarely been examined specifically in general urology inpatient populations, extending the relevance of our findings.

The association between ureteral interventions and AKI is supported by previous reports highlighting procedure-related ischemia, obstruction, infection, and contrast exposure as major contributors to postoperative renal injury [5,6,9].

Similarly, nephrolithiasis was also associated with increased AKI risk, likely reflecting the combined effects of obstruction, inflammation, and recurrent interventions. Interestingly, age was not independently associated with AKI in our cohort, in contrast to several surgical studies [10,12]. This may reflect the dominant influence of baseline kidney function and procedural factors in our population.

### 4.3. Methodological Considerations in AKI Definition

In this study, AKI was defined according to KDIGO creatinine-based criteria, without incorporation of urine output. This approach may have increased sensitivity for detecting mild or borderline AKI [4,12]. Previous urologic studies have used heterogeneous definitions, defining AKI as a postoperative increase in creatinine exceeding 2 mg/dL from baseline within 30 days [5] or using AKIN-based criteria [6], limiting comparability across cohorts.

Baseline creatinine was defined as admission creatinine because reliable outpatient data were unavailable. We did not stratify patients by creatinine-based estimated GFR using CKD-EPI, because standard eGFR equations are not reliable in non-steady-state conditions such as AKI and may misrepresent true kidney function when serum creatinine is dynamically changing [13]. Although elevated admission creatinine may represent underlying CKD, episodes of AKI superimposed on CKD are clinically meaningful and associated with adverse outcomes [1,2]. Our classification was therefore based on dynamic in-hospital changes, ensuring capture of both de novo AKI and AKI on CKD.

Monitoring-driven ascertainment bias should also be considered. Patients perceived as high risk underwent more frequent creatinine testing, potentially inflating AKI detection. Accordingly, the reported AKI incidence likely reflects a risk-enriched cohort and may overestimate the true incidence in the overall urology inpatient population. Because a formal comparison with non-included admissions was not feasible, the magnitude of this enrichment could not be quantified directly. This pattern is consistent with the risk of surveillance-related ascertainment bias in retrospective inpatient studies [14].

### 4.4. Clinical Outcomes and Prognostic Implications

Patients who developed AKI experienced significantly longer hospital stays, consistent with prior studies demonstrating that AKI prolongs hospitalization and increases healthcare costs [7]. Even transient AKI has been associated with adverse long-term outcomes, including CKD progression and mortality [1,7]. In our cohort, the association between AKI and prolonged hospitalization remained evident in complementary analyses, including non-parametric testing and outlier-sensitive analyses. Additional severity-oriented analyses showed that LOS was more pronounced among patients with more severe AKI; however, patients with Stage 1 AKI also had longer LOS than patients without AKI, supporting the clinical relevance of even mild kidney injury patterns. This difference should be interpreted cautiously, as a modest increase in length of stay may reflect greater overall clinical complexity and healthcare utilization rather than a large disease-specific effect on the original urologic diagnosis itself [1,7,15].

Because admission creatinine was higher in patients who developed AKI, part of the observed LOS difference may reflect reduced renal reserve and greater baseline clinical complexity rather than AKI alone. However, additional sensitivity analyses showed that the association between AKI and prolonged hospitalization persisted after restriction to patients with normal admission creatinine and remained directionally consistent after adjustment for admission creatinine, with significance retained in sensitivity analysis after exclusion of the extreme LOS outlier.

These findings suggest that both baseline renal vulnerability and superimposed AKI contribute to prolonged hospitalization. Importantly, elevated admission creatinine and pre-existing CKD should not be viewed solely as confounding factors to be statistically removed, as AKI superimposed on CKD is itself a clinically meaningful condition associated with adverse outcomes.

Despite longer hospitalization, no patients required dialysis and no in-hospital deaths occurred. This likely reflects the predominance of Stage 1 AKI and the limited sample size. Nevertheless, growing evidence indicates that mild AKI should not be regarded as benign [3,7], underscoring the importance of early detection. In the urology ward setting, AKI may often be associated with potentially reversible mechanisms such as urinary tract obstruction, peri-procedural hemodynamic changes, or post-obstructive diuresis [5,6,7,9,14]. Accordingly, the predominance of mild AKI in our cohort may represent an important window for timely recognition and supportive intervention.

### 4.5. Clinical Implications and Future Directions

Our findings have potentially relevant clinical implications, but these should be interpreted cautiously. In practical terms, they support earlier recognition of risk and more focused supportive prevention in selected hospitalized urology patients. This may include closer serum creatinine surveillance in higher-risk patients, careful review of potentially nephrotoxic medications, avoidance of unnecessary contrast exposure when feasible, and optimization of volume status and hemodynamic support according to the clinical setting.

These implications are consistent with the broader AKI literature, which emphasizes early recognition, prevention of further injury, and supportive management as the main currently actionable strategies in routine care [1,4]. Although most available interventional data derive from perioperative and other selected settings, they support the general principle that targeted surveillance and supportive prevention may be particularly relevant in patients at increased renal risk. Accordingly, our findings are best interpreted as supporting greater clinical vigilance and selective supportive measures in higher-risk urology inpatients, rather than as demonstrating a proven interventional pathway in this specific population.

The exploratory admission-based risk model developed in this study should be viewed as a tool for hypothesis generation and clinical awareness rather than as a stand-alone decision instrument. Its current role is to support early bedside risk stratification using simple admission variables, but not to direct definitive management decisions. This distinction is important because prediction modeling in AKI is an evolving field, and more recent models for general hospitalized patients have undergone external validation across independent hospital cohorts [16]. In contrast, our model was derived and evaluated within a single cohort and therefore requires both internal and external validation before any broader clinical application can be considered.

Future prospective studies should evaluate whether structured risk-based surveillance, supportive prevention strategies, and incorporation of broader clinical variables can improve prediction, reduce AKI-related complications, and strengthen clinical applicability in general urology inpatient populations. Future studies should also assess whether subgroup-specific models in more homogeneous surgical and non-surgical urology populations provide improved biological plausibility, calibration, and external validity.

### 4.6. Strengths and Limitations

This study has several strengths, including the use of standardized KDIGO criteria, comprehensive clinical and procedural data, and the development of an admission-based risk stratification model. The inclusion of both surgical and non-surgical patients broadens the descriptive relevance of the cohort and reflects routine inpatient urology practice; however, it also introduces mechanistic heterogeneity that may limit the biological specificity and generalizability of the exploratory prediction model.

Several limitations should be acknowledged. First, the retrospective design limits causal inference and is subject to information and selection bias. Second, urine output criteria were unavailable, which may have led to under-recognition of some AKI cases. Third, baseline creatinine was defined as admission creatinine due to limited availability of pre-admission data, potentially resulting in misclassification among patients with pre-existing CKD. In addition, we did not perform alternative baseline-definition analyses based on early in-hospital creatinine values, and therefore the sensitivity of the results to baseline choice could not be fully assessed. Moreover, because serum creatinine monitoring was clinician-driven rather than protocolized, the analytic cohort represents a monitored subgroup of urology inpatients, which may limit generalizability and may have led to overestimation of AKI incidence relative to the full ward population. In addition, because baseline data for the non-included admissions were not available in a sufficiently structured form for formal comparison, the magnitude of this surveillance-related selection bias could not be directly quantified, further limiting inference regarding the true incidence of AKI in the overall ward population.

Fourth, AKI etiology was not formally adjudicated, limiting mechanistic interpretation. Fifth, outcome assessment was restricted to short-term in-hospital measures, and post-discharge outcomes, including long-term renal recovery, CKD progression, and readmissions, were not available. In addition, several clinically relevant variables, including nephrotoxic medication exposure, contrast administration, fluid balance, and infection severity, were not collected in the retrospective dataset and therefore could not be evaluated as potential confounders or predictors.

Because the prediction model was derived and evaluated within the same single-center cohort, without formal internal resampling procedures such as bootstrapping or cross-validation, its performance estimates may be subject to optimism bias and potential overfitting. External validation in independent prospective cohorts is therefore required before broader clinical application, and future studies should also incorporate formal internal validation to assess model stability, calibration, and generalizability. In addition, because the cohort included both surgical and non-surgical admissions, the exploratory model was derived across clinically heterogeneous AKI mechanisms. We did not perform a stratified sensitivity analysis by hospitalization type, and therefore the stability and relevance of the model across these subgroups could not be assessed. In addition, ureteral stent placement and ureteroscopy were combined into a single procedural category in the exploratory model, which may have obscured procedure-specific contributions to AKI risk. Larger studies should evaluate these interventions separately.

## 5. Conclusions

In conclusion, AKI appears to be a frequent complication among monitored urology inpatients, particularly in those with higher clinical risks, and was associated with identifiable admission characteristics and prolonged hospitalization. Elevated admission creatinine, hypertension, nephrolithiasis, and ureteral interventions were major predictors of AKI development. Admission-based risk stratification may support early identification of vulnerable patients and facilitate targeted monitoring and supportive preventive strategies.

Although most AKI events were mild, supplementary analyses showed that even Stage 1 AKI was associated with longer hospitalization, supporting its clinical relevance. Further multicenter studies with extended follow-up and external validation are needed to confirm these findings and to determine whether structured, risk-based management approaches can improve long-term kidney and patient-centered outcomes.

## Figures and Tables

**Table 1:** Baseline characteristics and major comorbidities of patients with and without AKI.

	AKI (*n* = 67)	No AKI (*n* = 129)	*p*-Value
Age, mean ± SD (years)	65.9 ± 15.6	61.3 ± 19.4	0.0720
Male sex, *n* (%)	55 (82.1)	83 (64.3)	0.0156
Diabetes mellitus, *n* (%)	30 (44.8)	40 (31.0)	0.0564
Hypertension, *n* (%)	44 (65.7)	54 (41.9)	0.0026
Dyslipidemia, *n* (%)	33 (49.3)	49 (38.0)	0.1724
Congestive heart failure, *n* (%)	8 (11.9)	7 (5.4)	0.1790
Malignancy, *n* (%)	19 (28.4)	25 (19.4)	0.2119

*p*-value < 0.05 was considered statistically significant. AKI = acute kidney injury; SD = standard deviation.

**Table 2:** Multivariable logistic regression model for factors associated with AKI during hospitalization. The table presents adjusted odds ratios for candidate variables included in the multivariable model. A reduced exploratory admission-based risk model was subsequently derived from clinically available admission variables. AKI = acute kidney injury; OR = odds ratio; CI = confidence interval. A *p*-value < 0.05 was considered statistically significant.

Variable	OR	95% CI	*p*-Value
Admission creatinine (per 1 mg/dL)	3.1	1.8–5.5	<0.0001
Ureteral stent/ureteroscopy	2.9	1.3–6.7	0.01
Hypertension	2.5	1.2–5.2	0.02
Nephrolithiasis	2.2	1.1–4.5	0.03
Male sex	1.8	0.8–4.3	0.18
Age (per year)	1.0	0.9–1.1	0.91

## Data Availability

The data that support the findings of this study are not openly available due to reasons of sensitivity and are available from the corresponding author upon reasonable request. Data are in controlled-access data storage at the Institute of Nephrology and Hypertension, Barzilai University Medical Center, Ashkelon, Israel.

## Supplementary Materials

The following supporting information can be downloaded at: https://www.mdpi.com/article/10.3390/jcm15093495/s1, File S1: STROBE Checklist for Cohort Studies.
