# Supplementary material for: Risk Factors and Prediction of Acute Kidney Injury in Hospitalized Urology Patients: A Retrospective Cohort Study
Source: J Clin Med. 2026 May 2;15(9):3495. doi: 10.3390/jcm15093495 (PMC13163560; doi:10.3390/jcm15093495)
Supplement: Supplementary file 1 [file jcm-15-03495-s001.zip › jcm-4239454-supplementary.pdf]

Section/Topic	Item No.	STROBE recommendation	Page No.	Notes
Title and abstract	1a	Indicate the study's design with a commonly used term in the title or the abstract	1	Retrospective cohort design stated in title and abstract
Title and abstract	1b	Provide in the abstract an informative and balanced summary of what was done and what was found	1	Structured abstract
Introduction	2	Explain the scientific background and rationale for the investigation being reported	2	Introduction background and rationale
Introduction	3	State specific objectives, including any prespecified hypotheses	2	Primary and secondary objectives stated
Methods	4	Present key elements of study design early in the paper	2	Retrospective observational cohort study
Methods	5	Describe the setting, locations, and relevant dates, including periods of recruitment, exposure, follow-up, and data collection	2	Hospital, country, and study period stated
Methods	6a	Give the eligibility criteria, and the sources and methods of selection of participants. Describe methods of follow-up	2-3, 5	Eligibility criteria in Methods; cohort selection in Figure 1
Methods	6b	For matched studies, give matching criteria and number of exposed and unexposed	N/A	Not a matched study
Methods	7	Clearly define all outcomes, exposures, predictors, potential confounders, and effect modifiers. Give diagnostic criteria, if applicable	3-4	AKI definition, outcomes, predictors, and model variables described
Methods	8	For each variable of interest, give sources of data and details of methods of assessment (measurement). Describe comparability of assessment methods if there is more than one group	2-3	EMR and laboratory data sources described
Methods	9	Describe any efforts to address potential sources of bias	3-4	Selection/surveillance bias and baseline-definition limitations discussed
Methods	10	Explain how the study size was arrived at	3	No formal sample size calculation was performed; the cohort included all eligible admissions during the study period
Methods	11	Explain how quantitative variables were handled in the analyses. If applicable, describe which groupings were chosen and why	4	Continuous variables, cutoffs, and risk groups described
Methods	12a	Describe all statistical methods, including those used to control for confounding	4	Statistical tests and multivariable logistic regression described
Methods	12b	Describe any methods used to examine subgroups and interactions	4	Supplementary severity-oriented and restricted analyses described
Methods	12c	Explain how missing data were addressed	NR	Missing-data handling not explicitly reported
Methods	12d	If applicable, explain how loss to follow-up was addressed	N/A	No longitudinal follow-up beyond index hospitalization
Methods	12e	Describe any sensitivity analyses	4	Sensitivity analyses described
Results	13a	Report numbers of individuals at each stage of study—e.g., numbers potentially eligible, examined for eligibility, confirmed eligible,	5	Numbers screened, excluded, and analysed reported

Section/Topic	Item No.	STROBE recommendation	Page No.	Notes
		included in the study, completing follow-up, and analyzed		
Results	13b	Give reasons for non-participation at each stage	5	Exclusions summarized in Figure 1
Results	13c	Consider use of a flow diagram	5	Figure 1 provided
Results	14a	Give characteristics of study participants and information on exposures and potential confounders	6-7	Baseline characteristics shown in Table 1
Results	14b	Indicate number of participants with missing data for each variable of interest	NR	Not explicitly reported variable-by-variable
Results	14c	Summarize follow-up time (e.g., average and total amount)	N/A	In-hospital retrospective cohort; no longitudinal follow-up time metric
Results	15	Report numbers of outcome events or summary measures over time	8-11	AKI incidence, stage distribution, LOS, discharge outcomes, and model outputs reported
Results	16a	Give unadjusted estimates and, if applicable, confounder-adjusted estimates and their precision; make clear which confounders were adjusted for and why they were included	10-11	Baseline comparisons and adjusted ORs in Table 2
Results	16b	Report category boundaries when continuous variables were categorized	9-11	Risk-group thresholds and normal admission creatinine cutoff reported
Results	16c	If relevant, consider translating estimates of relative risk into absolute risk for a meaningful time period	N/A	Not applicable to this exploratory logistic model
Results	17	Report other analyses done—e.g., analyses of subgroups and interactions, and sensitivity analyses	9-11	Restricted, outlier, and severity-oriented analyses reported
Discussion	18	Summarize key results with reference to study objectives	11	Principal findings section
Discussion	19	Discuss limitations of the study, taking into account sources of potential bias or imprecision. Discuss both direction and magnitude of any potential bias	15	Strengths and limitations section
Discussion	20	Give a cautious overall interpretation of results considering objectives, limitations, multiplicity of analyses, results from similar studies, and other relevant evidence	11-14	Discussion sections 4.1–4.5
Discussion	21	Discuss the generalizability (external validity) of the study results	15	Limitations and future directions address generalizability
Other information	22	Give the source of funding and the role of the funders for the present study and, if applicable, for the original study on which the present article is based	17	No external funding

Supplementary File S1. STROBE Checklist for Cohort Studies. N/A = not applicable; NR = not explicitly reported. Checklist wording follows the STROBE recommendations for cohort studies.
